# “Freezing” Thermophiles: From One Temperature Extreme to Another

**DOI:** 10.3390/microorganisms10122417

**Published:** 2022-12-06

**Authors:** Tetyana Milojevic, Margaret Anne Cramm, Casey R. J. Hubert, Frances Westall

**Affiliations:** 1 Exobiology Group, CNRS-Centre de Biophysique Moléculaire, University of Orléans, Rue Charles Sadron, CEDEX 2, 45071 Orléans, France; 2 Geomicrobiology Group, Department of Biological Sciences, University of Calgary, 2500 University Drive NW, Calgary, AB T2N 1N4, Canada; 3 Exobiology Group, CNRS-Centre de Biophysique Moléculaire, Rue Charles Sadron, CEDEX 2, 45071 Orléans, France

**Keywords:** thermophiles, low temperature, cold environments

## Abstract

New detections of thermophiles in psychrobiotic (i.e., bearing cold-tolerant life forms) marine and terrestrial habitats including Arctic marine sediments, Antarctic accretion ice, permafrost, and elsewhere are continually being reported. These microorganisms present great opportunities for microbial ecologists to examine biogeographical processes for spore-formers and non-spore-formers alike, including dispersal histories connecting warm and cold biospheres. In this review, we examine different examples of thermophiles in cryobiotic locations, and highlight exploration of thermophiles at cold temperatures under laboratory conditions. The survival of thermophiles in psychrobiotic environments provokes novel considerations of physiological and molecular mechanisms underlying natural cryopreservation of microorganisms. Cultures of thermophiles maintained at low temperature may serve as a non-sporulating laboratory model for further exploration of metabolic potential of thermophiles at psychrobiotic temperatures, as well as for elucidating molecular mechanisms behind natural preservation and adaptation to psychrobiotic environments. These investigations are highly relevant for the search for life on other cold and icy planets in the Solar System, such as Mars, Europa and Enceladus.

## 1. Introduction

**Figure 1:**
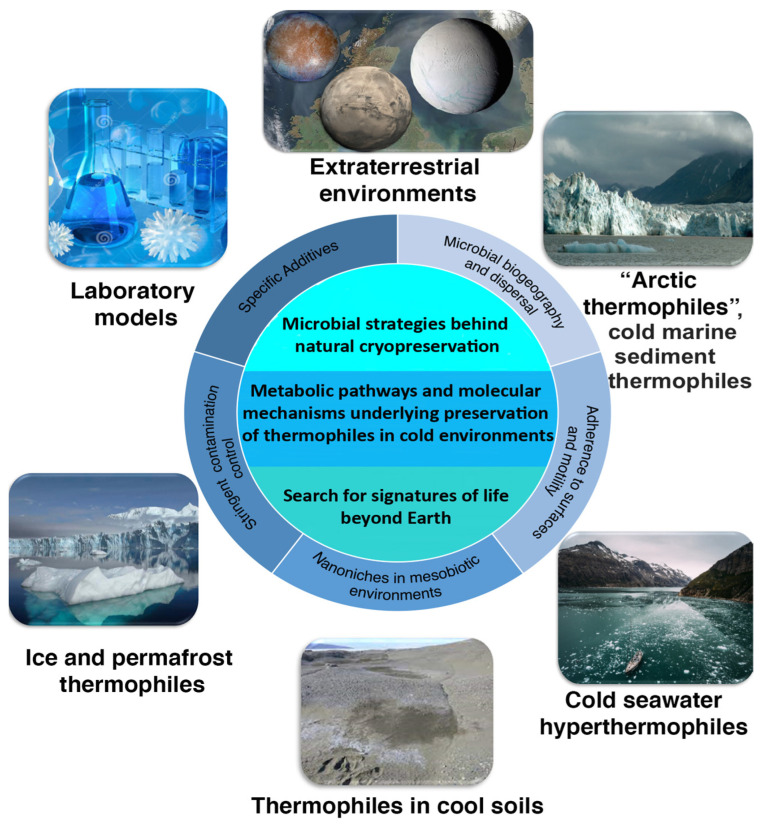
A conceptual framework for comprehensive assessment and understanding of the presence of thermophiles in cold marine and terrestrial habitats.

A variety of extremophilic microorganisms have been found in environments characterized by parameters extending beyond the range of their physiological activity [1,2], such as thermophilic bacterial endospores in low temperature marine settings [3]. The occurrence of thermophiles (organisms which grow above 40 °C) in psychrobiotic environments has been described in several independent investigations (Figure 1, Table 1). Thermophiles detected in cold marine and terrestrial habitats span several phyla across the tree of life, with both Bacteria and Archaea (Table 1). Recent 16S rRNA gene sequencing studies have uncovered a number of putative thermophiles in icy [4,5,6,7] and permafrost environments [8,9,10,11], permanently cold (−2 °C to +4 °C) ocean sediments [12,13,14,15,16,17,18,19], and in cool soils with temperatures never exceeding 25 °C [20,21,22,23,24,25] (Figure 1).

Thermophilic spores in cold marine sediments have been explored in detail as models for understanding passive dispersal and biogeography. They comprise >10% of the total endospore population in permanently cold sediments from Aarhus Bay [27] and their dormancy enables their rate of dispersal into the cold seabed to be estimated [3]. Spores are specialized cellular structures that protect cells from what would be lethal stresses such as radiation, desiccation, toxins, and extreme heat or cold, and allow the cells to withstand periods of nutrient starvation [42,43]. Spore formation can contribute to the survival of thermophiles in Arctic sediments through dormancy [9,26]. In soils, thermophilic spores are found in abundances of >1.5 × 10^4^ per gram [20,35]. It is intriguing how these thermophilic populations withstand psychro- and mesobiotic environments where temperatures are significantly below their minimum requirement for growth [25,44]. Typically, minimum temperatures reported for enabling growth and metabolic activity of thermophiles are obtained from experimental data using relatively short incubations and artificial culture conditions that differ from environmental conditions that the microorganisms were isolated from. Laboratory growth conditions are not normally designed to detect thermophiles at lower temperatures. The experimental data suggest that the extreme thermoacidophilic archaeon *Metallosphaera sedula* withstands cold stress and can be maintained at low temperature, when cultivated with multimetallic mineral material as the sole energy source [41]. These observations, after their further thorough analysis and validation, may open an avenue to investigate strategies and underlying molecular mechanisms of survival of thermophilic species in psychrobiotic environments (Figure 1).

Furthermore, unraveling the thermal limits of microbial life and the survival limits of thermophiles in low temperature habitats is also decisive for envisaging the microbial survival during transfer through space and between celestial bodies, e.g., in the context of lithopanspermia. Microbial “space travelers” are subject to extreme temperature fluctuation during their interstellar transit [45,46]. Microbial cryopreservation studies are also helpful in terms of defining possible spatial and temporal near-surface microenvironments suitable for microbial life beyond Earth, e.g., on Mars, where microorganisms may be protected not only against harsh radiation, but also from extreme temperature fluctuations (Figure 1). Recently, microniches in the Martian regolith containing chaotropic ions that could favor microbial growth and preservation at low temperature and thus support the potential Martian biosphere [47], have been proposed by Chin et al. [48]. Moreover, comprehensive investigations into the survival limits of thermophiles in low temperature habitats may add new knowledge on ancestral thermophiles with simpler and smaller genomes in cool primordial environments and complement the on-going scientific debates concerning the psychrophilicity or thermophilicity of a last universal common ancestor [49,50,51,52,53].

### 1.1. “Arctic Thermophiles” and Cold Seawater Hyperthermophiles

The discovery of thermophiles in any cold environment is always a somewhat peculiar event, and observations of these misplaced microbes are downplayed in polar habitats. Thermophilic spore-forming bacteria (“thermospores”) in the marine sediments of Svalbard, at almost 80° North, have been reported on different occasions [3,19,26]. This is consistent with a long history of thermophiles detections in sediments with in situ temperatures too cold for their growth [12,13,36,54]. They remain dormant and inactive at the cold temperatures at which they are found and are detected only after pasteurization and high-temperature incubation of marine sediment. Arctic thermospores in Svalbard fjord sediments typically grow at 45–65 °C. Research has focused on their occurrence as spores and how this dormancy mechanism equips these bacteria for long-term viability in psychrobiotic environments as members of the microbial seed bank and rare biosphere [55,56].

As dormant spores, these thermophiles offer models for studying microbial biogeography and dispersal. Psychrobiotic environments maintain the dormant phenotype of thermophilic spores and in this state, they are not influenced by factors typically shaping biogeography that rely on microbial activity and growth, such as environmental selection, drift and mutation [19]. Thus, the dormant phenotype of thermophilic endospores enables focused investigations of passive microbial dispersal, which is another factor shaping microbial biogeography. For example, rates of dispersal can be quantified by evaluating the number of non-growing spores in a sediment depth profile in relation to the sediment accumulation rate in that location. This is shown through high temperature germination experiments by Hubert et al. [3], where a stable abundance of thermophilic spores (10^5^ g^−1^ sediment) with depth was combined with the sedimentation rate (determined by ^210^Pb decay to be 0.2 cm y^−1^ in this Svalbard fjord) pointing to a constant flux of thermophilic spores into the seabed at a deposition rate of 10^8^ m^−2^ y^−1^. Using a similar high temperature incubation approach, de Rezende et al. [15] determined a comparable thermospore deposition rate (10^7^ m^−2^ y^−1^) in Baltic Sea sediment, and furthermore identified some of the same thermophile phylotypes as those being deposited in the Svalbard fjord sediments, despite >3000 km distance between the two locations. At a finer geographic scale, Arctic thermophilic spores enriched from marine sediment from 10 different fjord sites along the west coast of Spitsbergen, Svalbard showed biogeographic patterns suggesting different warm-to-cold dispersal histories and source environments for different taxa [19]. Conversely, return of thermospores from cold to hot environments through sediment burial has been suggested by Heuer et al. [57] and Gittens et al. [58]. These studies highlight the utility of thermophilic spores for exploring the temporal and lateral extent of microbial dispersal.

These observations have led to new hypotheses about dispersal vectors linking warm habitats to the cold ocean, and the fluxes needed to facilitate such dispersal. Several studies have shown that marine microbial diversity and biogeography are influenced by ocean currents [59,60,61,62]. These influences determine distributions of marine microorganisms in general, including thermophilic spores, and imply that microbial propagules can be transported long distances via connected water bodies. Thermophilic spores have also been detected in the water column (through incubating filters at high temperature) above the cold marine sediments in which they have been studied [15,27] suggesting passive transport from seawater into the seabed via sedimentation. Particulate transport from the water column into the sediments is often facilitated by what are known as marine flocs or marine snow. This could serve as a vector for transport of microbial propagules. Furthermore, Müller et al. [16] identified an association between the connectivity of a marine sediment sites to the global ocean circulation and the diversity and richness of its thermophilic spore-forming populations. Areas more connected to global ocean currents show a greater diversity of thermophiles, and these connected sites share a greater number of thermospore phylotypes in common.

The dispersal of themophilic spores into cold sediments (Table 1, Figure 1) raises questions about their origins, i.e., the warm habitat(s) that they must be supplied from. In the examples above, a recurring result of 16S rRNA gene and other marker gene sequence analyses have shown that cold sediment thermophiles are often most closely related to *Clostridia* from warm sub-marine deep biosphere habitats (e.g., petroleum reservoirs and mid ocean ridge systems) [3,14,15,16,17,63]. Typically, several taxonomic groups of thermophilic spores are identified in a given location, indicating the possibility that multiple warm source habitats are seeding thermophilic spores into the ocean and eventually into the studied cold ocean sediments [19]. Similarly, thermophiles found in terrestrial cold environments may be dispersed via atmospheric currents, as has been speculated by Ziegler [25]. In this way, studies of thermophiles existing as dormant microbial propagules in permanently cold environments hint at underlying mechanisms of microbial passive dispersal that contribute to marine microbial diversity, and are separate from confounding factors of environmental selection, drift, and mutation. While these dispersal mechanisms may be identified through the study of thermospore biogeography, the mechanisms themselves may influence and contribute to marine microbial diversity beyond the dormant microbiome. Additionally, activity assays that retain taxonomic identity, such as meta-transcriptomics and genomic-based stable isotope probing, should be used to investigate whether low-level psychrotolerant activity by spore-forming thermophiles occurs, and whether the presence and activity of thermophiles shapes the active microbial community in cold marine sediment. Along with spores of thermophiles detected in psychrobiotic and mesobiotic marine sediments, spreading and long-term survival of non-spore-forming hyperthermophiles at relatively low temperatures (2 to 4 °C) in cold, oxic seawater has also been reported [33,64] (Table 1, Figure 1). Thermophilic and hyperthermophilic microorganisms (e.g., belonging to the genus *Thermococcus*) have been cultured from 3–30 °C hydrothermal fluids in the northeastern Pacific Ocean [28]. Laboratory studies show that hyperthermophiles (various *Thermococcus*-species) can survive at least 9 months in cold surroundings when stored in low-temperature seawater [30]. Hyperthermophilic methanogens have been isolated from low-temperature (20–34 °C and 6 °C) hydrothermal fluids of Axial Volcano and Endeavour Segment in the northeastern Pacific Ocean [29]. Furthermore, hyperthermophilic *Thermococcales* show long-term survival at low temperatures (4 °C), which is important for the wide distribution, dispersal, and dominance of these archaea at deep-sea hydrothermal vents [31]. The presence of hyperthermophilic microorganisms in low-temperature vent fluids emanating from the seafloor suggests that they originate from a subseafloor hydrothermal habitat where temperatures are within their growth range. It has been proposed that hyperthermophiles present in low abundance in cold seawater in a hibernation-like state, are incidentally transferred to the outside of black smokers and actively occupy specific niches in chimney walls, where conditions are optimal for their physiology [32].

The dispersal of hyperthermophilic microorganisms via cold seawater (Table 1, Figure 1) raises questions about how these heat-loving non-sporulating cells can withstand conditions detrimental to their physiological requirements. Adherence to surfaces and motility have been suggested as factors promoting long-term survival of black smoker-associated hyperthermophiles in cold seawater [30,32]. The exact mechanism of microbial colonization of newly formed black smokers still remains to be investigated experimentally. The capabilities for thermo- and chemotaxis enable cells to quickly react to encountering high temperatures, e.g., by means of very fast motility the cells swim in the direction of the warm temperature, moving from very cold to hot environments. After reaching an optimal temperature region they can “explore” the surface via a zigzag-movement and use their motility to establish contact, adhering with their flagella at a suitable place and forming biofilms. Wirth (2017) [32] proposes that additional adhesins may contribute to permanent colonization of black smokers. In this way, studies of these cold seawater hyperthermophiles are revealing possible passive dispersal mechanisms facilitating global connectivity of marine habitats important for microbial distribution.

### 1.2. Thermophiles in Cool Soils

The presence of high numbers of thermophilic bacteria in cool soil environments has been shown for subsurface soil samples from different continents [20,21,22,23,24,25]. Following enrichment at 70 °C and 80 °C, five highly thermophilic aerobic bacteria were isolated from cool soil environments in Northern Ireland and Bolivia [20]. The thermophilic isolates showed high growth rates at 70 °C, some with doubling times shorter than 30 min, and were characterized by unusual cellular morphology, including long flexible cells as well as short rods. Based on 16S rRNA gene sequence analysis, these isolates are closely related to *Geobacillus thermoglucosidasius* and an unclassified member of the thermophilic geobacilli (98%) [20]. The isolation of 53 thermophilic, aerobic, spore-forming and non-spore-forming bacteria was reported for the subsurface soil environment in Northern Ireland [24]. The majority were affiliated with *Geobacillus thermoleovorans* (n = 27) or *Geobacillus caldoxylosilyticus* (n = 18). Several isolates exhibited only 93% similarity with *Geobacillus toebii* strain F70, thus likely representing new species of *Geobacillus*. Interestingly, closely related bacteria are widely distributed in other soil environments, suggesting that—contrary to the thermospores in cold marine sediments—the soil thermophiles might not be introduced from an external source, but are rather autochthonous members of these environments [24]. Alternatively, an estimated thermophile input rate of 1.4 × 10^5^ cells m^−2^ yr^−1^ in Irish soils has been suggested, sustaining a viable population of thermophiles in cold temperate environments. Plate counting suggests that these thermophiles may comprise up to 10% of the total cultivable aerobic bacteria in cool soils (1.5–8.8 × 10^4^ colony forming units g^−1^) [22,23]. Dispersal may be facilitated by continuous transfer into soils via cloud droplets, providing a constant supply of thermophiles into these environments [22,23].

The isolation of thermophilic bacteria from cool soils has elicited a research interest concerning the possible metabolic activity by these microorganisms in conditions that differ from their optimum requirement for growth (Figure 1). Survival of a representative cool soil isolate, *Geobacillus thermoleovorans* strain T80, was investigated at low temperatures, revealing an increase in its population during a long-term (9-month) cultivation at 4 °C [22]. This increase was attributed to very slow growth rates accompanied by negligible rates of cell death. Interestingly, cultures of *G. thermoleovorans* maintained at temperatures slightly higher (25 °C) but still below the organism’s growth-permissive thermal range, did not show a similar population size increase. The increase of cell density only at 4 °C appears to reflect a preservation mechanism for suppressing cell death. Microcosms spiked with *G. thermoleovorans* also showed that the surviving viable geobacilli could persist at low temperatures after 1 week of cultivation [22].

The hypothesized existence of nanoniches in mesobiotic environments has been proposed by Wiegel et al. [65] based on similar observations of anaerobic alkalithermophiles isolated from mesobiotic environments that grow faster than those isolates from thermobiotic habitats. It was proposed that transient niche establishments in these environments provide short-term limited conditions for alkaliphilic thermophilic bacteria and therefore enable elevated growth rate in order to cope with this limitation [65]. At the molecular level, it has been proposed that microorganisms capable of growth at temperatures considered both thermobiotic and mesobiotic may encode different sets of key enzymes whereby gene expression and protein synthesis of either set is regulated by temperature [40].

Investigating microbiological populations within Icelandic basaltic and rhyolitic glass and minerals (−20.1–44.5 °C), researchers detected putative thermophiles as a minor component of microbial communities of these volcanic rocks [66]. Once again, these microbial species are atypical for a sub-Arctic environment where the temperature does not approach the range required for thermophile activity. *Geobacillus* and *Thermobacterium* sequences have been identified via G2 PhyloChip-assisted microarray analysis of 16S rRNA gene amplicons from the whole rock community genomic DNA at this site [34,67]. Seven thermophiles belonging to the genus *Geobacillus* were isolated from the rock Icelandic community, with several exhibiting a minimum growth temperature of 36 °C [66]. These thermophiles may be dispersed from Icelandic hot springs or from aerosols originating outside Iceland. Year-long measurements of the temperatures in crystalline basalt and obsidian glass at 2 cm depth reveal that the thermal conductivity of the rocks was sufficient to transiently allow for temperatures above 36 °C, up to 44.5 °C. The low albedo of these rocks has been suggested to facilitate their thermal conductivity such that the near-surface environment of these igneous materials can support transient growth of thermophiles in summer months [66]. Icelandic thermophiles are proposed to transiently contribute to local biogeochemical processes, since their microclimatic environment inside the rock pores provides a certain potential for their temporary “re-awakening” during the warm periods of the year. Therefore, similar to the aforementioned *Geobacillus* isolates in cool soils, these thermophiles may be indigenous inhabitants, not necessarily exogenous propagules, and could perform ecologically relevant functions. In this way they may be similar to thermophilic bacteria found in temperate soils that participate in C, N, S, and P cycling [68,69] and treatment of wheat crops with thermophilic *Ureibacillus* which benefits the crops by reducing toxic metals and increasing plant stress tolerance [70].

### 1.3. Ice and Permafrost Thermophiles

Accretion and glacial ice of Lake Vostok is another permanently cryobiotic environment where molecular signatures for thermophilic life have been reported (Table 1). Underlying a 4 km thick Antarctic ice sheet, the subglacial Lake Vostok represents an extreme environment with pressure approaching 400 atmospheres, an ultra-low nutrient supply from the glacier melt, water temperature averaging −2 °C, and a possible excess of oxygen and nitrogen [4,5]. This environment was assumed to be lifeless until DNA-based assays suggested that this is a unique ecosystem supporting a diverse microbiota, but at cell abundances lower than in most environments on Earth [71]. Thermophiles have been detected in samples of Lake Vostok accretion ice, i.e., naturally frozen lake water accreted to the glacier floating above the lake. Microbial life resident in the subglacial Lake Vostok is represented by the facultative chemolithoautotroph *Hydrogenophilus thermoluteolus* (*Proteobacteria*, class *Hydrogenophilalia*), a non-spore-forming thermophile that has also been isolated from hot springs. Initially, *H. thermoluteolus* was detected in a sample of a Lake Vostok borehole drilled at a depth of 3607 m [5]. Later, *H. thermoluteolus* has been recognized with high confidence as one 16S rRNA bacterial phylotype with relevance to the lake environment [4]. The authors applied stringent decontamination techniques and contaminant database criteria, performing molecular analyses in two different laboratories. The results of 16S rRNA gene sequencing have been confirmed by targeted analysis of *cbbL*/*rbcL* (encoding RubisCO in most chemoautotrophs) and *hox*/*hya*/*frh*/*hup* (encoding a NiFe hydrogenase) genes from *H. thermoluteolus* in the same samples [4]. More recent investigations of Lake Vostok ecology based on metagenomic and metatranscriptomic analyses of accretion ice, has uncovered the presence of additional putative thermophiles residing in both types of accretion ice [7]. Naturally, the presence of drilling fluid traces in polar ice cores raises the important question of contamination during sampling, representing a real challenge for associated molecular biological studies. As an approach to address this contamination issue, the analysis of the drilling fluids obtained from a Lake Vostok borehole has been performed [72]. The 16S rRNA gene amplicon sequencing of samples derived from the drilling fluids used during the ice core retrieval process [72] revealed dominated putative hydrocarbon-degrading organisms and less represented populations that cannot metabolize hydrocarbons. Alekhina et al. [72] proposed that all the bacteria present in these drilling fluid samples must be considered as contaminants in the context of Lake Vostok analyses. However, *Hydrogenophilus* spp. were not detected in drilling fluids samples. The very low organic carbon and nutrient contents in accretion ice of Lake Vostok suggests that conditions favor chemolithoautotrophic life. The existence of deep faults at the bottom of the lake has been proposed, where hydrogen would permit chemolithotrophs such as *H. thermoluteolus* to flourish. Indeed, Lake Vostok is a tectonically controlled subglacial lake with recently recorded minor tectonic activity. The hydrothermal plumes caused by episodic seismotectonic activity in deep faults in the bedrock of the lake could release sediments along with microbial cells from faults towards vents in the freezing zone, where the microbes may have been entrapped in the ice in course of accretion [4,5,6]. The cultivation and recovery of viable *Hydrogenophilus* spp. from the accretion ice would offer direct evidence that the frozen cells in Lake Vostok can be revived.

A number of thermophilic and thermotolerant chemolithoautotrophs that use inorganic sources of energy (e.g., hydrogen, nitrogen, sulfur, or iron) to support their metabolism have recently been detected by metatranscriptomic and metagenomic approach in samples of accretion ice originated from the embayment region of Lake Vostok (3563 + 3585 m) [37]. The large number of unique and total sequences of these microorganisms was found to be in line with increases in concentrations of nutrients and microelements (amino acids, organic carbon, Na^+^, Ca^2+^, Mg^2+^, Cl^−^ and SO_4_^2−^). The authors suggest that the influence of the hydrothermal activity located several kilometers away, or the presence of saltwater in this region (e.g., a brine layer) may contribute to a high number of thermophilic and thermotolerant organisms. Further metaproteomic and metabolomic profiling of these environmental samples might be helpful in order to characterize the activity of thermophiles in such icy environments.

Fascinating hydrothermal springs that occur in the continuous permafrost zone have described to harbour thermophilic microbial communities [73]. Thermophilic species in the permafrost of Kamchatka peninsula and Deception Island have been revealed among microbial communities of frozen sedimentary deposits [8,9,10,11] (Table 1, Figure 1). These studies were primarily conducted by the research group led by the late David Gilichinsky, a leading permafrost scientist who focused on microbial preservation in ancient ice. Mironov et al. [10] reported the presence of a thermophilic community in frozen volcanic ash and scoria. In this study, hyperthermophiles were isolated from a borehole in the volcanic deposits of Deception Volcano. These cultures were shown to utilize sulfur as an electron acceptor and grow optimally at 85–95 °C. Mironov et al. [10] described thermophilic methanogens, acetogens and sulfate reducers growing on CO_2_ and H_2_ that were enriched from permafrost in the Kamchatka peninsula. Optimal growth between 65 and 75 °C was observed for these cultures, including one that shares 100% rRNA gene sequence identity to extremely thermophilic bacteria of the genus *Caldicellulosiruptor* [10]. A group of moderate thermophiles isolated from Antarctic permafrost (six surface and subsurface-isolated isolates) has been also previously described [8]. Mironov et al. [39] reported on thermophilic non-spore-forming bacteria of the genus *Geobacillus* in permafrost samples from volcanic sedimentary rocks of the Deception Island. Naturally, the isolation of viable thermophiles and hyperthermophiles raised questions about the origin of these microorganisms and their lifestyle in such environments. It was suggested that some groups of anaerobic thermophilic bacteria, associated with volcanic activity, might have survived in permafrost over long periods, becoming embedded and stored in frozen sediments [9]. The authors considered a volcanic eruption or surrounding strata associated with volcanoes as possible sources of thermophiles that were isolated from permafrost [9]. Microbial airborne dispersal is most probably facilitated by sedimentation of ashes, pyroclastic material, and atmospheric precipitation [9]. Thermophilic microbial communities in permafrost regions (Table 1) may represent the most ancient storage and persistence of thermophiles preserved in the frozen state.

Applying cultivation techniques and 16S rRNA gene sequencing, a recent study reported the presence of culturable thermophiles along the extreme temperature gradient in Deception volcano [38]. Thermophilic members of the genera *Geobacillus*, *Brevibacillus*, *Anoxybacillus*, *Thermus*, and *Bacillales* order were recovered from both fumaroles and glaciers, with environmental temperatures between 80 and 0 °C. The majority of these described recovered thermophilic isolates in the Deception glaciers (0 °C, Table 1) were spore-forming bacteria with a good culturability and a pronounced resistance towards desiccation and UV-C irradiation [38]. The authors proposed the coexistence and a potential interplay between thermophiles and psychrophiles in polar volcano Deception. The results of this work indicate that these thermophiles remain viable even when environmental conditions are outside their growth range [38].

### 1.4. Laboratory Models for the Exploration of Thermophiles in Cold Environments

Our understanding of the genetics and physiology of how thermophiles adapt to the cold is still in its infancy. Consequently, physiological capacities, metabolic pathways and molecular mechanisms underlying preservation of thermophiles in cold environments have not been thoroughly investigated. Molecular approaches targeting global gene expression, proteomics and metabolomics profiles could deliver an enhanced view of the specific adaptation of thermophilc life to low temperatures. The major limitation in this field comes from a restricted cultivation of thermophiles at low temperature regiment underlaboratory conditions. The rare exception is the *G. thermoleovorans* strain T80, which was cultivated at 4 °C during a long-term period (9-month) and in soil microcosms at low temperatures during short-term experiments (1 week) [22].

Wiegel [40] described that extreme thermophile *Methanobacterium thermoautotrophicum* can grow between 22 and 78 °C and the addition of sterile anaerobic sediments permitted its incubations at lower temperatures. Moreover, *M. thermoautotrophicum* strain JW 501 was capable of steady methane production at 15 °C over several months, suggesting some metabolic activity under non-growth permissive temperature [40].

**Figure 2:**
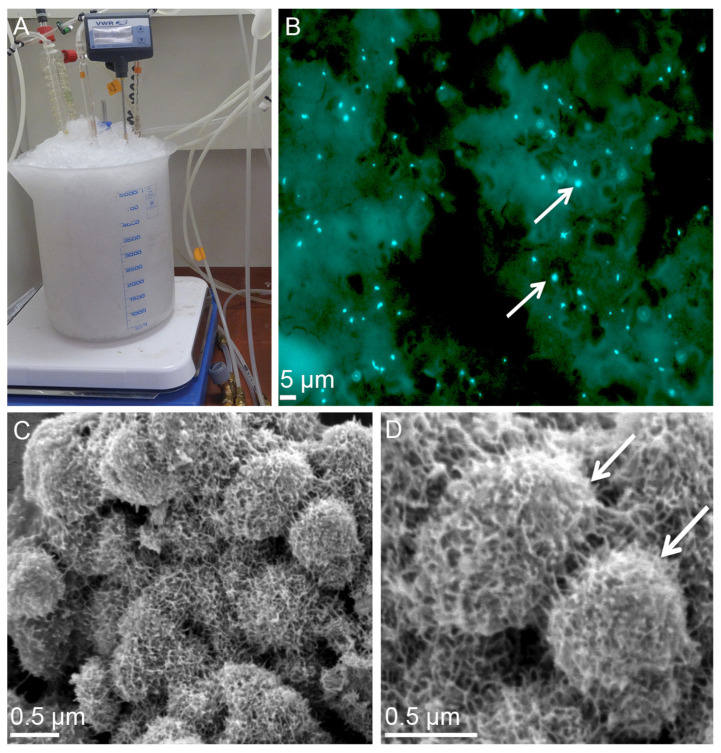
The cultivation of *M. sedula* cells with the stony meteorite NWA 1172 as the sole energy source at the cold temperature regime. (**A**) The cultures of *M. sedula* at low temperature in 1 L glassblower modified Schott-bottle bioreactors. *M. sedula* cultures were incubated at the average temperature of 12 °C in an ice-supplemented environment (modified from Milojevic et al. [41]). (**B**) The multi-labeled fluorescence in situ hybridization (MiL-FISH) of *M. sedula* cells supplemented with the stony meteorite NWA 1172 as the sole energy source at low temperature. An overlaid epifluorescence image shows overlap of specific probe with DAPI signals (modified from Milojevic et al. [26]). Cultures of *M. sedula* were examined with MiL-FISH [78,83] following 2 months of cultivation at 12 °C. (**C**) A scanning electron microscopy (SEM) image of *M. sedula* supplemented with the stony meteorite NWA 1172 as the sole energy source at low temperature. (**D**) A higher magnification SEM image displaying single cells of *M. sedula* covered with extracellular matrix. Cells of *M. sedula* are indicated by the white arrows. Cultures of *M. sedula* were examined with SEM after 2 months of cultivation at the average temperature of 12 °C.

The extreme thermoacidophile *Metallosphaera sedula* is among thermophiles supported at cold temperatures under the laboratory conditions (Table 1, Figure 2). *M. sedula* is a facultative chemolithotroph and metal-mobilizing archaeon, which respires iron and reduced inorganic sulfur compounds [74,75,76,77,78,79,80,81,82]. *M. sedula* is known as an obligate thermophile with a temperature range from 50 to 80 °C permissive for growth and an optimum of 73 °C [83,84]. It is worth noting, that *M. sedula* was first isolated from the sediments of Pisciarelli solfatara (Naples, Italy), where the temperature was much lower, between 25 and 52 °C [83]. Interestingly, none of the energy sources that the organism uses at higher temperatures (pyrite, chalcopyrite, and other inorganic electron donors) are able to support the cultivation of *M. sedula* in this lower temperature range [83,84]. The only exception is the chemolithoautotrophically grown *M. sedula* cultivated at low temperatures (Figure 2) [41] in the presence of preliminary powdered and sterilized multimetallic meteorite material (the stony chondrite H5 type NWA1172 [81,85]) as the sole energy source. Supplementation with NWA 1172 permitted the cultivation of *M. sedula* during two months at the average temperature of 12 °C [41]. The presence of *M. sedula* cells in these experiments was confirmed by multi-labeled fluorescence in situ hybridization (MiL-FISH) with an *M. sedula*-specific ribosomal RNA-targeted probe [81,86], as well as sequencing of the *M. sedula* specific marker gene *msed0966* (Figure 2B) [41]. Interestingly, cold-maintained *M. sedula* cells were characterized by a branched extracellular matrix on the cell surface (Figure 2C,D). This layer of cellular appendages wrapping around the colonies of *M. sedula*, together with a tendency to clump and condense into cellular aggregates, may represent a possible adaptive strategy for withstanding cold conditions [41]. *M. sedula* cells maintained at low temperature thus offer a laboratory model to investigate metabolic potential of non-spore-forming extreme thermophiles in cold environments, microbial strategies, and molecular mechanisms behind natural cryopreservation.

In similar work, Chin et al. [48] also proposed that specific additives such as chaotropic metabolites and generally chaotropic environments can increase tolerance to subzero temperatures, extending growth windows for microbial survival in cold environments. Taken together, several studies indicate that the utilization of specific additives, including energy substrates and/or nutrients, e.g., the addition of sterile anaerobic sediments [40], genuine soil microcosms [22], and metal-rich mineral material [41], can dramatically influence the resistance to cold stress, increasing the preservation of thermophiles in the cold.

### 1.5. Potential for Thermophiles in Extraterrestrial Environments

In terms of astrobiology and the search for extraterrestrial life, the existence of thermophiles in cold environments is of great interest. Mars was habitable in the past, having liquid water, organic molecules and sources of energy (redox gradients, heat, radiation) [87]. Indeed, investigations of the Curiosity rover in Gale Crater revealed that the lake waters had a relatively neutral pH, as documented by the smectite clays, minerals, such as magnetite, Fe–sulphates and –sulphides, compounds indicating variable redox states, and the relatively long-lived presence of water, all suggesting the existence of habitable conditions about 3.6 Ga [47,88]. Moreover, recent investigations have detected organic molecules in situ [89,90,91]. Mars has also provided the essential palette of biological building blocks: energy sources, Carbon, Hydrogen, Nitrogen, Oxygen, Phosphorous and Sulfur (CHNOPS) elements, as well as important catalytic transition metals associated with life, as we know it. However, while traces of past life may be found at the surface of the ancient terranes of Mars, the objectives of the Mars Science Laboratory and the Curiosity rover [47], the Mars 2020 mission [92], and the ExoMars 2022 mission with its Rosalind Franklin rover [93], extant life, if it still exists, will be in the subsurface. Mars is very cold; its surface may be compared with permafrost and its subsurface aquifers are most likely frozen. However, in the very recent past (~50,000 y) volcanic activity has occurred [94]. Molten lavas rising through the cryosphere and permafrost could create habitable niches with temperatures ranging from thermobiotic to the psychrobiotic, thus permitting cold-preserved cells to revive and metabolise and, possibly, reappear briefly at the surface of the planet with any volcanically-associated hydrothermal fluids.

The icy satellites, Europa and Enceladus, orbiting around Jupiter and Saturn, respectively, are considered to be habitable and potentially inhabited [95,96,97,98]. Mobility of the icy crust and plumes of volatiles (water, salts and organic molecules (the latter for Enceladus)) [99,100,101] ejected from their surfaces testify to the presence of liquid water below the icy shells. In both cases, the presence of a fractionated core and mantle, plus tidal heating due to gravitational pull from the parent planets, suggest the possibility of hydrothermal activity at the rock/water interface at the bottom of the oceans. Missions, such as Europa Clipper and the proposed Europa Lander [102,103], aim to search for signatures of life in the plumes and/or icy crust of Europa. If life exists on either of these two icy bodies, it may well be some form of thermophilic life form, that could be trapped in the ice (as at Lake Vostok) or expelled in the plumes.

### 1.6. Molecular Targets to Address Mechanisms of Survival of Thermophiles in Psychrobiotic Environments

The metabolic and molecular mechanisms that could allow viability and even minimal metabolic activity of thermophiles in cold environments are largely unknown and unexplored. Thermophilic life forms have been reported in a variety of natural environments with different degree of human influence ranging from such areas as Lake Vostok with no human habitation to soil environments close to settlements with a high index of anthropogenic influence (Table S1). Apparently, anthropogenic activity can be excluded as a factor affecting the distribution of thermophiles in cold settings. At the same time, the analysis of extremophilic properties of the reported thermophiles in Table 1 shows that “freezing” thermophiles are polyextremophilic microorganisms capable of tolerating several environmental extremes (Table 2). Apart from heat resistance, predominant majority of these thermophiles have metal tolerance or even resistance and require no oxygen for their lifestyle. Almost a half of these thermophiles can live on the account of inorganic carbon source (CO_2_), frequently in extreme pH conditions and in salt rich environments. Some of them are radiation tolerant species and several can withstand or even flourish in high pressure conditions (Table 2). “Freezing” thermophiles in Table 1 are almost equally represented by both spore-forming and non-spore-forming species. Hence, the physiological and molecular mechanisms of their adaptation to cold environments might go beyond the formation of endospores. Other extremophilic properties summarized in Table 2 (e.g., resistance against metals, pH extremes, radiation, high pressure and salinity) can also contribute to the explanation of the presence of these organisms in cold settings. Further focusing on the mechanisms of the maintenance and persistence of thermophilic microorganisms in psychrobiotic environments may lead to the development of new concepts of microbial physiological plasticity.

The molecular strategies that enable these microbes to retain viability beyond their growth range still remain to be substantially discovered. Here, among prospective directions for the future research is a pull of respiratory enzymes such as for instance high affinity NiFe-hydrogenases. This family of microbial enzymes is responsible for scavenging of atmospheric gases and providing a sufficient energy input for long-term persistence in stress-inducing conditions [135]. Among the unusual properties of these enzymes are oxygen insensitivity and a broad thermal stability window (−4 °C to +60 °C) [135]. The insensitivity of these enzymes to environmental stress factors, such as temperature, might also corroborate energy production that takes place even under unfavorable and hostile conditions [135]. Microbial mesophilic isolates from temperate forest and agricultural soils exhibited high-affinity H_2_ uptake due to the presence of these enzymes [136] that can be activated in response to energy limitation to persist in the absence of microbial growth [135]. The thermoacidophilic methanotroph *Methylacidiphilum fumariolicum* SolV is capable of gas (e.g., H_2_ and CO_2_) fixation [137] under moderate temperatures employing both oxygen-sensitive and -insensitive high-affinity NiFe-hydrogenases [138,139]. This area of enzymes of energy metabolism in thermophiles along with investigations of the minimal energetics required for maintenance of thermophiles viability in psychrobiotic environments is of great potential for future research prospects to address knowledge gaps in molecular strategies of “freezing” thermophiles. As for non-spore-forming thermophiles, the focus of future research might concentrate on a mineral protection crust [80,81,82], which augments the structural stability of prokaryotic cell envelops [140] and can protect their cells from harsh extracellular environment, including stress with cold temperatures [41] and desiccation [134]. Apart from it, natural cryoprotectants (e.g., trehalose, prolyne, glycine betaine, and any other yet to be discovered), which thermophilic spore-forming bacteria and prokaryotic non-spore-formers produce to preserve their membrane integrity during freezing, are among other interesting targets for further research in this area. All these above-mentioned potential molecular strategies of survival are presented in Table S2 as potential targets for the future research on “freezing” thermophiles.

## 2. Materials and Methods

A review of the occurrence of thermophilic microorganisms in psychrobiotic environments worldwide (cold marine sediments, cold seawaters, ice, permafrost, cool soils, etc.), and in laboratory conditions was performed. Articles published, with no restriction on language and with no date restrictions, were included, according to the requirements of the Preferred Reporting Items for Systematic Reviews and Meta-Analyses (PRISMA) [141]. PubMed/MEDLINE, Web of Science, Scopus, and Google Scholar databases were used in all cases. The keywords used were “thermophiles”, “cold temperature(s)”, “cold temperature(s)”, “permafrost” and “ice”. In addition, the bibliographies of the included papers were screened to incorporate additional sources that might have been overlooked during the primary database search. The results included microbiological studies with review and research articles, with case reports being included. The articles were chosen using the following inclusion criteria: worldwide studies reporting direct detection of thermophiles in one of the environments of interest (cold marine sediments, cold seawaters, ice, permafrost, cool soils, laboratory culture media, etc.) with full text available. The following exclusion criteria were used: methodological studies aiming only to the development or improvement of microbial detection methods, studies performed in warm or hot environments, studies without available full text, and duplicates.

## 3. Conclusions

The presence of thermophilic microbes in psychrobiotic environments (Figure 1, Table 1) has been known for many years [12]. The biogeographic patterns of their dispersal and distribution have been intensively discussed [15,16,20,21,24] and unexpected high abundances and dispersal rates of spore-forming thermophiles into psychrobiotic environments have been reported [3,15]. A general graphical representation of all psychrobiotic environments with detected thermophiles along with some of their physiological strategies for survival there is given in Figure 1. At the same time, the origin, dispersal, supply and preservation mechanisms of non-spore-forming thermophiles native to hyper- and thermobiotic habitats in mesobiotic/psychrobiotic environments remain enigmatic. Our understanding of the molecular machinery and physiology of non-spore-forming thermophiles in response to conditions of psychrobiotic/mesobiotic environments remains limited mainly due to a failure to support cultivation of thermophilic species in artificial culture media at low temperatures. Supplementation with specific additives (e.g., multimetallic mineral materials [26] or soil microcosms [22,68] may offer further possibilities to study the physiological and molecular mechanisms implicated in the survivability of non-spore-forming thermophiles at deep subfreezing temperatures. Importantly, future investigations should deliver a systematic study to determine which constituents of these materials are the crucial ingredients to ensure thermophiles culture preservation at low temperatures.

Novel insights into the physiology of thermophiles in cold environments (Figure 1) can also be gained from the functional studies of thermophiles maintained at cold temperatures by means of targeting specific genes, examining global gene expression using microarrays for mRNA levels and total intracellular and extracellular proteins and metabolites using proteomic and metabolomic profiling (Table S2). Additional focus on membrane lipids, particularly for strains such as *M. sedula* and *G. thermoleovorans*, under different growth temperatures, will also be important. These kinds of insights can be combined with biophysical and biochemical studies to investigate the structural and functional properties of protein machinery implicated in the preservation of thermophiles at low temperatures (Table S2). Apart from state-of-the-art molecular techniques, it is important to emphasize that future research strategies should be directed towards incubation of thermophiles under simulated or/and in situ environmental conditions in order to minimize any possible discrepancies arising from artificial culture conditions. Such a multi-disciplinary approach can dramatically enhance our understanding of unique ways that certain thermophiles evolved adapting to low temperature environments. Furthermore, thermophilic microorganisms are widely known for their biotechnological potential, e.g., in the industrial production of bio-based chemicals, renewable biofuels, nutrition supplements, fertilizers, polymeric substances, biologically active compounds with antiviral, antiseptic, and anticancer activities, bioremediation and biomining operations, to name only a few major ones. Operating some of these bioprocesses over a wide range of temperatures might certainly boost bioprocessing by growing or maintaining thermophiles under low temperatures. Low temperature regiment of the selected thermophiles might be an attractive target from the side of metabolic engineering, yielding the increased survival rates in cold areas. Finally, increased understanding of thermophiles in psychrobiotic environments is essential in aiding the search for past, or extant life, on other planets, such as Mars or some of the icy satellites orbiting Jupiter and Saturn.

## Figures and Tables

**Table 1:** “Freezing” thermophiles in cold environments.

Microorganisms	Growth Temperature Ranges	Psychrobiotic Environments	Temperature of Psychrobiotic Environments	References
“Arctic thermophiles” and other “cold marine sediment thermophiles”				
Thermophilic *Desulfotomaculum* spp., *Caminicella* sp., *Caloranaerobacter* sp.	45 to 64 °C	marine surface sediment, Smeerenburgfjorden, Svalbard	−2 to +4 °C	[3,14]
*Desulfotomaculum* spp.	50 °C	marine surface sediment, west Spitsbergen, Svalbard	−2 to +7 °C	[19]
*Desulfotomaculum arcticum*	26 to 46.5 °C	marine sediment, Nordfjorden, Svalbard	+1.8 °C	[26]
*Desulfotomaculum geothermicum*, *D. halophilum*, *D. thermosapovorans*, *D. arcticum*, *D. kuznetsovii*, *D. alkaliphilum*, *D. aeronauticum*, *D. australicum*	46 to 69 °C	marine surface sediment, Aarhus Bay	0 to 15 °C	[13]
115 thermophilic *Firmicutes* primarily affiliated to *Clostridiales*, *Bacillales*, *and Thermoanaerobiales orders*	50 °C	marine surface sediments from 111 deep water location in Eastern Gulf of Mexico	not available	[17]
146 thermophilic phylotypes affiliated to the *Bacillales*, and *Clostridiales* orders	50 °C	marine surface sediment from 81 locations around the world’s ocean	0 to 50 °C	[16]
*Geobacillus stearothermophilus*	65 °C	marine sediment from basins of the continental slope from Ensenada, Mexico to Santa Catalina Island	~4 °C	[12]
Thermophilic bacteria primarily affiliated with *Tepidibacter Defluvialea*, *Bacillus*, and *Clostridiisalibacter* genera	50 °C	marine seawater and surface sediment, Aarhus Bay, Denmark	max. 15 °C	[27]
*Desulfotomaculum kuznetsovii*	50 to 70 °C	marine sediment, Aarhus Bay, Denmark	max. 15 °C	[13]
*Thermosediminibacter oceani*, *Thermopsediminibacter litoriperuensis*	52 to 76 °C	deep sea sediments, Peru Margin, eastern equatorial Pacific	Not available	[14]
Cold seawater hyperthermophiles				
Thermophilic and hyperthermophilic microorganisms *Hydrogenimonaceae*, *Aquificales* families	45 to 95 °C	open ocean seawater	3–30 °C	[28]
Hyperthermophiles, various *Thermococcus*-species	55 to 98 °C	low-temperature seawater	6 °C	[29]
Thermophilic and hyperthermophilic microorganisms, the genus *Thermococcus*	55 to 98 °C	hydrothermal fluids in the northeastern Pacific Ocean	3–30 °C	[30]
Hyperthermophilic methanogenes *Methanocaldococcus* spp.	48 to 94 °C	low-temperature hydrothermal fluids of Axial Volcano and Endeavour Segment, northeastern Pacific Ocean	20–34 °C, 6 °C	[31]
Hyperthermophilic archaea of the order *Thermococcales*	55 to 98 °C	diffuse hydrothermal vents, the northeast Pacific Ocean	4 °C	[32]
Hyperthermophiles *Pyrodictium*, *Pyrococcus*, *Thermococcus*, *Archaeoglobus* spp.	60 to 110 °C	open-sea waters next to Macdonald Seamount	4 °C	[33]
Thermophiles in cool soils				
Unclassified thermophilic bacillus, *Geobacillus thermoglucosidasius*	40 to 80 °C	cool soil environments in Northern Ireland and Bolivia	max. 25 °C	[20]
53 thermophilic, aerobic, spore-forming and non-spore-forming bacteria; *Geobacillus thermoleovorans* *Geobacillus caldoxylosilyticus*	optimum 70 °C	subsurface soil environment in Northern Ireland	max. 25 °C	[24]
Thermophilic bacteria *Geobacillus* spp. *Thermobacterium* spp.	36 to 65 °C	Icelandic basaltic and rhyolitic glass and minerals, sub-Arctic environments	−20.1–44.5 °C	[34]
*Geobacillus stearothermophilus*	25 to 70 °C	Icelandic soil	max. 27 °C	[35]
Thermophilic bacteria	55 °C	Soil, Point Barrow, Alaska	max. ~20 °C	[36]
Ice and permafrost thermophiles				
Facultative chemolithoautotroph, thermpophile from hot springs *Hydrogenophilus thermoluteolus*	50 to 87 °C	Subglacial Lake Vostok’s accretion ice, water	−2 °C	[5]
Thermophiles	50 to 87 °C	Subglacial Lake Vostok’s accretion ice, water	−2 °C	[4,7]
Thermophilic and thermotolerant species: *Thauera hydrothermalis*, *Desulfurella acetivorans*, *Hydrogenophilus thermoluteolus*, *Bacillus* sp., *Thermobispora bispora*, *Anoxybacillus flavithermus*, *Thermosynechococcus* sp., *Caldimonas sp.*, *Gordonia* sp., *Geobacillus* sp., *Amphibacillus* sp.	25 to 87 °C	Accretion ice originated from the embayment region of Lake Vostok	below 0 °C	[37]
Thermophilic community, Hyperthermophiles	55 to 95 °C	Frozen volcanic ash and scoria, the volcanic deposits of Deception Volcano	−2 °C	[10]
Thermophilic members of the genera *Geobacillus*, *Brevibacillus*, *Anoxybacillus*, *Thermus*, and *Bacillales*	35 to 79 °C	Glaciers, Deception Volcano	0 °C	[38]
Thermophilic methanogens, acetogens and sulfate reducers growing on CO_2_ and H_2_; extremely thermophilic bacteria of the genus *Caldicellulosiruptor*	optimum 65 to 75 °C	Permafrost in the Kamchatka peninsula	−2 °C	[10]
Moderate thermophiles	-	Antarctic permafrost	max. 0 °C	[8]
Thermophilic non-spore-forming bacteria of the genus *Geobacillus*	37 to 78 °C	Permafrost from volcanic sedimentary rocks of the Deception Island	−2 °C	[39]
Laboratory models for the exploration of thermophiles in cold environments				
Extreme thermophile *Methanobacterium thermoautotrophicum*	78 °C	Several months of laboratory cultivation with the addition of sterile anaerobic sediments	15 °C	[40]
*Geobacillus thermoleovorans strain* T80	50–75 °C	Long-term (9-month) laboratory cultivation with soil microcosms	4 °C	[22]
Extreme thermoacidophile *Metallosphaera sedula*	50–80 °C	2 months of laboratory cultivation in the presence of sterile multimetallic meteorite material (stony chondrite H5 type NWA1172)	12 °C	[41]

**Table 2:** Extremophilic properties of “freezing thermophiles” presented in Table 1.

Thermophile	Heat Resistance	Metal Tolerance	Radiation Resistance	Pressure Resistance	Desiccation Resistance	pH Extremes	Autotrophy	O_2_	Spore	Salt Tolerance	Ref.
*Desulfotom-aculum* spp.	+	+	+	+	+	+	+	−	+	+	[104,105]
*Caminicella* spp.	+	N/A	N/A	N/A	N/A	N/A	−	−	+	+	[106]
*Caloranaer-obacter* spp.	+	+	N/A	N/A	N/A	N/A	−	−	+	+	[107]
*Bacillales*	+	+	+	+	+	+	−	+/−	+	+	[108,109]
*Thermoana-erobiales*	+	N/A	N/A	N/A	N/A	+	−	−	+	N/A	[110,111]
*Geobacillus* spp.	+	+	+	+	+	+	+/−	+/−	+	−	[112]
*Tepidibac-ter*	−	+	+	N/A	+	N/A	−	−	+	+	[113,114]
*Clostridiisa-libacter*	−	N/A	N/A	N/A	N/A	N/A	−	−	+	+	[115]
*Thermosedi-minibacter*	+	+	N/A	N/A	N/A	N/A	−	−	−	−	[54]
*Thermo-coccus* spp.	+	+	+	+	N/A	+	−	−	−	+	[116,117]
*Methano-caldococcus* spp.	+	+	N/A	N/A	N/A	−	+	−	−	−	[118]
*Pyrodictium*	+	+	N/A	+	N/A	−	+	−	−	+	[119]
*Pyrococcus*	+	+	+	+	N/A	−	−	−	−	N/A	[120,121,122]
*Archaeo-globus*	+	+	+	+	+	N/A	+/−	−	−	+	[123]
*Hydrogeno-philus thermo-luteolus*	+	N/A	N/A	N/A	N/A	−	+	+	−	N/A	[124]
*Thauera hydrother-malis*	+	N/A	N/A	N/A	N/A	+	−	−	−	N/A	[125]
*Desulfurella acetivorans*	+	+	N/A	N/A	N/A	+	+/−	−	−	N/A	[126]
*Thermobispora bispora*	+	N/A	N/A	N/A	N/A	N/A	−	+	+	N/A	[127]
*Anoxybacillus flavi-thermus*	+	+	+	N/A	N/A	−	+	−	+	N/A	[128]
*Thermosyne-chococcus* spp.	+	N/A	N/A	N/A	N/A	N/A	+	+	+	N/A	[129]
*Caldimonas* spp.	+	+	N/A	N/A	N/A	−	−	+/−	−	N/A	[130]
*Caldicellu-losiruptor*	+	+	N/A	N/A	N/A	−	−	−	−	N/A	[131]
*Methanobacterium thermoauto-trophicum*	+	+	−	+	N/A	+	+	−	−	−	[132,133]
*Metallo-sphaera sedula*	+	+	+	N/A	+	+	+	+	−	N/A	[77,80,134]

## Data Availability

Data supporting reported results can be found in Supplementary Materials.

## Supplementary Materials

The following supporting information can be downloaded at: https://www.mdpi.com/article/10.3390/microorganisms10122417/s1, Table S1: Some properties of psychrophilic habitats of thermophilic microorganisms; Table S2: Main strategies and molecular targets to address molecular mechanisms of survival of thermophiles in psychrobiotic environment.
