# Supplementary material for: “Freezing” Thermophiles: From One Temperature Extreme to Another
Source: Microorganisms. 2022 Dec 6;10(12):2417. doi: 10.3390/microorganisms10122417 (PMC9782878; doi:10.3390/microorganisms10122417)
Supplement: Supplementary file 1 [file microorganisms-10-02417-s001.zip › microorganisms-1974991-supplementary.pdf]

Table S1. Some properties of psychrophilic habitats of thermophilic microorganisms.

Environment	Man-made or nature environment	Human influence	Temperature (Table 1)
Svalbard, marine surface sediments	nature environment	low to very low human influenced*	-2 to +7°C
Aarhus Bay	nature environment	Close to settlements with high human influenced index	0 to 15°C
Eastern Gulf of Mexico, marine surface sediments	nature environment	Close to settlements with high human influenced index	not available
Mexico to Santa Catalina Island, marine surface sediments	nature environment	Close to settlements with high human influenced index	~4°C
Deep sea sediments, Peru margin, eastern equatorial Pacific	nature environment	Close to settlements with middle to high human influenced index	not available
Axial Volcano and Endeavour Segment, northeastern Pacific Ocean	nature environment	Close to settlements with middle to high human influenced index	6–34 °C
Macdonald Seamount	nature environment	low to very low human influenced	4 °C
Cool soil environments in Northern Ireland and Bolivia	nature environment	Close to settlements with high human influenced index	max. 25°C

Environment	Man-made or nature environment	Human influence	Temperature (Table 1)
Subsurface soil environment in Northern Ireland	nature environment	Close to settlements with high human influenced index	max. 25°C
sub-Arctic environments	nature environment	low to very low human influenced	-20.1-44.5 °C
Point Barrow, Alaska	nature environment	low human influenced	max. ~20°C
Lake Vostok	nature environment	very low human influenced	below 0 °C, -2°C
Deception Volcano	nature environment	very low human influenced	0°C
Kamchatka peninsula	nature environment	low human influenced	-2°C

*Wildlife Conservation Society - WCS, and Center for International Earth Science Information Network - CIESIN - Columbia University. 2005. Last of the Wild Project, Version 2, 2005 (LWP-2): Last of the Wild Dataset (Geographic). Palisades, New York: NASA Socioeconomic Data and Applications Center (SEDAC). https://doi.org/10.7927/H4348H83. Accessed 20 Nov 2022.

Table S2. Main strategies and molecular targets to address molecular mechanisms of survival of thermophiles in psychrobiotic environments

Mechanisms of thermo- and chemotaxis
Respiratory enzymes (NiFe-hydrogenases) & other enzymes of energy metabolism
Enzymatic machineries regulated by different temperatures
Investigations of the minimal energy required for survival in cold environments
Natural cryoprotectans
Interactions of cell surface molecules with metals and mineral particles
Proteomic and metabolomic profiling, lipidomics, transcriptomics, genomic studies
Biophysical/biochemical structural and functional studies of proteins implicated in cryopreservation
